# Biceps brachii muscle hardness assessed by a push-in meter in comparison to ultrasound strain elastography

**DOI:** 10.1038/s41598-020-77330-5

**Published:** 2020-11-20

**Authors:** Mitsuyoshi Murayama, Kazunori Nosaka, Takayuki Inami, Norihiro Shima, Tsugutake Yoneda

**Affiliations:** 1 Institute of Physical Education, Keio University, 4-1-1, Hiyoshi, Kouhoku-ku, Yokohama, Kanagawa 223-8521 Japan; 2 Centre for Exercise and Sports Science Research, School of Medical and Health Sciences, Edith Cowan University, Joondalup, WA 6027 Australia; 3 Department of Sport and Health Science, School of Sport and Health Science, Tokai Gakuen University, Miyoshi, Aichi 470-0207 Japan; 4 Department of Physiology, School of Health and Sports Science, Juntendo University, Inzai, Chiba 270-1695 Japan

**Keywords:** Physiology, Health care

## Abstract

This study investigated the relationship between push-in meter (PM) and ultrasound strain elastography (USE) for biceps brachii (BB) muscle hardness. BB hardness of 21 young men was assessed by PM and USE during rest and isometric contractions of six different intensities (15, 30, 45, 60, 75, 90% of maximal voluntary contraction: MVC) at 30°, 60° and 90° elbow flexion. Muscle hardness (E) was calculated from the force–displacement relationship in PM, and strain ratio (SR) between an acoustic coupler (elastic modulus: 22.6 kPa) and different regions of interest (ROIs) in BB was calculated and converted to Young’s modulus (YM) in USE. In resting muscle, E was 26.1 ± 6.4 kPa, and SR and YM for the whole BB was 0.88 ± 0.4 and 30.8 ± 12.8 kPa, respectively. A significant (*p* < 0.01) correlation was evident between E and logarithmical transformed SR (LTSR) for the ROI of whole BB (r = − 0.626), and E and converted YM (r = 0.615). E increased approximately ninefold from resting to 90% MVC, and E and LTSR (r = − 0.732 to − 0.880), and E and converted YM for the SR above 0.1 were correlated (r = 0.599–0.768, *p* < 0.01). These results suggest that muscle hardness values obtained by PM and USE are comparable.

## Introduction

Muscle hardness is defined as the resistance of muscle tissue against deformation by an applied force to the muscle, and is used as a parameter to evaluate muscle property^1,2^. Muscle hardness is often assessed by palpation subjectively, but it does not provide its value. To quantify muscle hardness, a push-in meter (PM) that measures the indentation distance and force by pressing a probe from the body surface to a muscle has been used^2–5^. Previous studies using a PM showed that muscle hardness was increased by muscle contraction^6,7^, muscle spasm or spasticity^8,9^, muscle damage induced by eccentric exercise^4^ and compartment syndrome^5^.

Muscle hardness can be also assessed by ultrasound strain elastography (USE), which provides visual and quantitative assessment of mechanical properties of a tissue^10^. In USE, an operator manually compresses an ultrasound transducer against the surface of a target muscle, and an elastogram is constructed based on the principle that softer tissue has more deformation, therefore indicates larger strain, in comparison to harder tissue^11^. The strain of the muscle is expressed as strain ratio (SR), which is the ratio of the relative strain between the muscle area and a reference area (e.g., muscle/acoustic coupler). SR has been used as an index of muscle hardness, and when SR is less than 1.0, it shows that the muscle is harder than the reference object. The use of USE for muscle hardness assessment has been increasing^11–13^. However, it has not been systematically investigated whether muscle hardness assessed by PM and USE is comparable.

In both PM and USE, the measurement principle in evaluating the relationship between the force and displacement is the same. Therefore, it is reasonable to assume that a high correlation is found in muscle hardness measures by the two methods. In fact, three studies compared between a hand-held PM and USE, and reported that the muscle hardness changes before and after exercise or myofascial release therapy assessed by the two methods were similar^14–16^. However, the correlation coefficient of the muscle hardness values between PM and USE was not reported in the studies. Furthermore, there are many types of hand-held PM on the market, and many of them induce a relatively small amount of indentation^17,18^, thus their validity is not necessarily confirmed. To evaluate muscle hardness, it is necessary to account for the influence of the pressure from the skin on subcutaneous tissue. We have developed a PM system that can indent a muscle over 20 mm, separate force–displacement data into subcutaneous tissue and muscle components, and calculate the elastic component of the muscle as a Young's modulus (YM)^3^. However, no previous study has compared the muscle hardness expressed by YM obtained by the PM system and the strain ratio by the USE.

The aim of this study, therefore, was to examine the relationships between PM and USE for muscle hardness measures of resting and contracting biceps brachii muscle. It was hypothesized that the muscle hardness values for resting and contracting biceps brachii obtained by PM (Young’s modulus) and USE (SR) would be significantly correlated.

## Methods

### Participants

Twenty-one healthy men (average ± SD age: 29.6 ± 7.8 years, height: 176.6 ± 7.4 cm, body mass: 76.8 ± 8.5 kg, biceps brachii muscle thickness: 25.5 ± 3.9 mm) participated in Experiment 1 (resting muscle condition), and 16 of them also participated in Experiment 2 (contracting muscle condition). The physical characteristics of the 16 participants in Experiment 2 were similar to those of the 21 participants in Experiment 1. This study was approved by the Human Ethics Committees of Keio University and Edith Cowan University. All methods were carried out in accordance with relevant guidelines and regulations. All participants were informed of the purpose, examination procedures and the potential risk of the study, and signed an informed consent.

### Study design

#### Experiment 1: Resting condition

**Figure 1:**
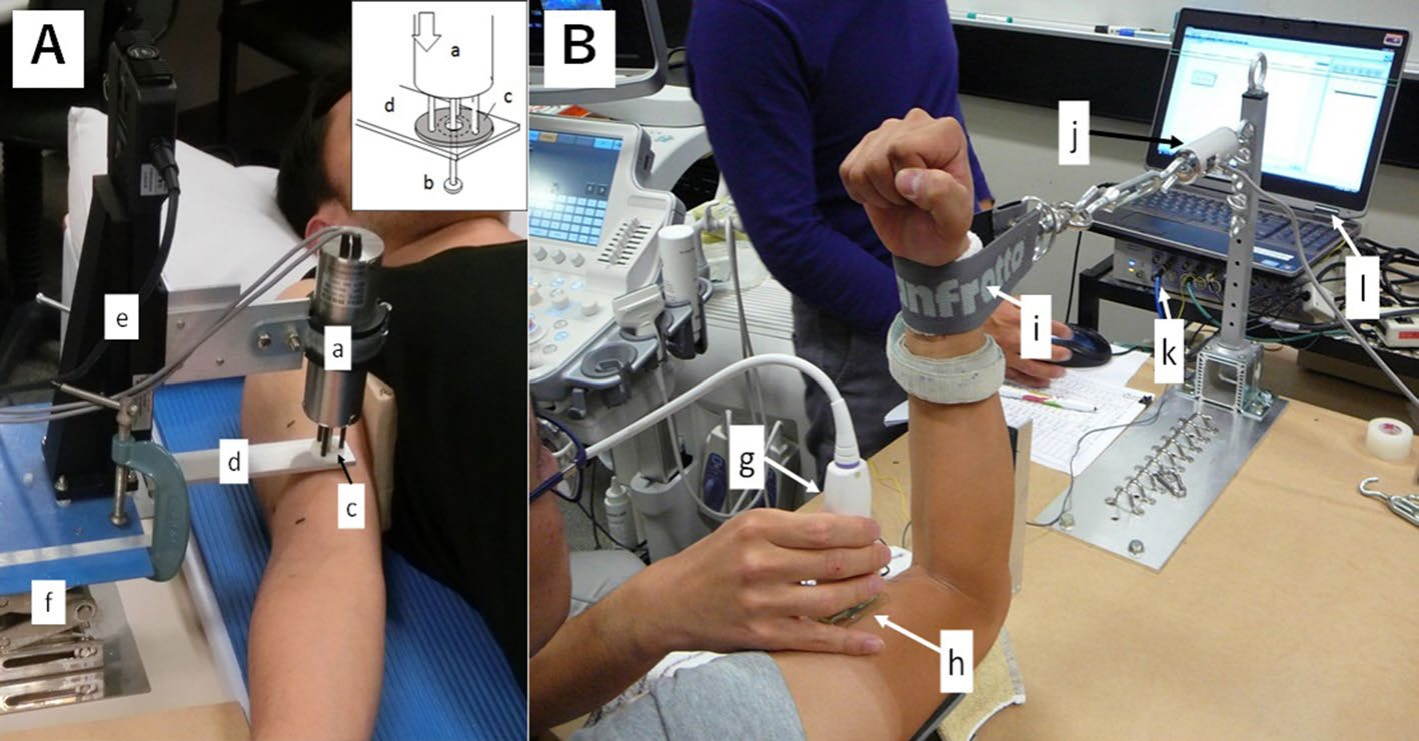
Set-up for the push-in meter (PM) assessment of muscle hardness in resting condition (**A**) and for the ultrasound strain elastography (USE) assessment of muscle hardness in contracting condition at 90° elbow angle as an example (**B**). (**A**) The inset shows the detail of the probe of PM. The cylindrical body of PM (a) has an un-movable probe (ϕ = 10 mm, length = 30 mm) with a force transducer (b) and a movable sensor plate for displacement detection (c). PM was attached to a z-axis stage (e) which controlled by the stage control unit. The stage was set on a jack (f), and PM was set on a stopping bar (d) fixed to the jack. A hole on the stopping bar only allowed the probe to enter. Moving the stage caused the probe to depress and push the underlying tissue while the sensor plate was pushed back by the stopping bar, detecting tissue displacement. In this system, 20-mm pushing into the tissue was performed from the skin surface. Each participant lied on his back on the table and the elbow kept at an elbow extended position (0°). (**B**) A probe (g) of USE is placed on the muscle belly with an acoustic coupler (h) in between. The investigator manually compressed the muscle by the probe. A strap (i) is attached to the wrist that is connected to a force transducer (j) with a turnbuckle. The force transducer is connected to a data acquisition system (PowerLab) (k) via a computer (l). Each participant was provided the visual feedback of their performed force on a computer screen simultaneously. The same set-up of a participant as that is shown in A was used for the USE measure for the resting muscle, and the same set-up of a participant as that is shown in B was used for the PM measure too.

Each participant lay on his back on a massage table with relaxing both arms. The elbow joint was kept at an extended position (0°) with a support (Fig. 1A). The muscle hardness measurement was taken at biceps brachii muscle belly of the right upper arm. Using B-mode ultrasound image (Prosound F75; Hitachi Aloka Medical, Japan), the point where the biceps brachii muscle thickness was the largest was assessed and marked by a pen. Muscle hardness measurements by a push-in meter and ultrasound strain elastography were taken from the same point.

#### Experiment 2: Muscle contraction condition

Each participant sat on a chair and the right upper arm in a pronation position was placed on a table (Fig. 1B). The measurement point was the same as that of Experiment 1. The output force by the contraction of the elbow flexors was recorded by a load cell (LUR-A-SA1, Kyowa, Japan) connected to the wrist of each participant by a strap with a turnbuckle (Fig. 1B). The load cell was connected to a strain amplifier (AS2103, NEC Sanei, Japan) which was connected to a PowerLab system (ADInstruments, Australia) controlled by a Labchart software (ADInstruments, Australia) installed in a computer (HP pavilion 15, HP Japan Inc., Japan). The force was displayed on a computer screen in order to provide the necessary visual feedback to each participant.

**Figure 2:**
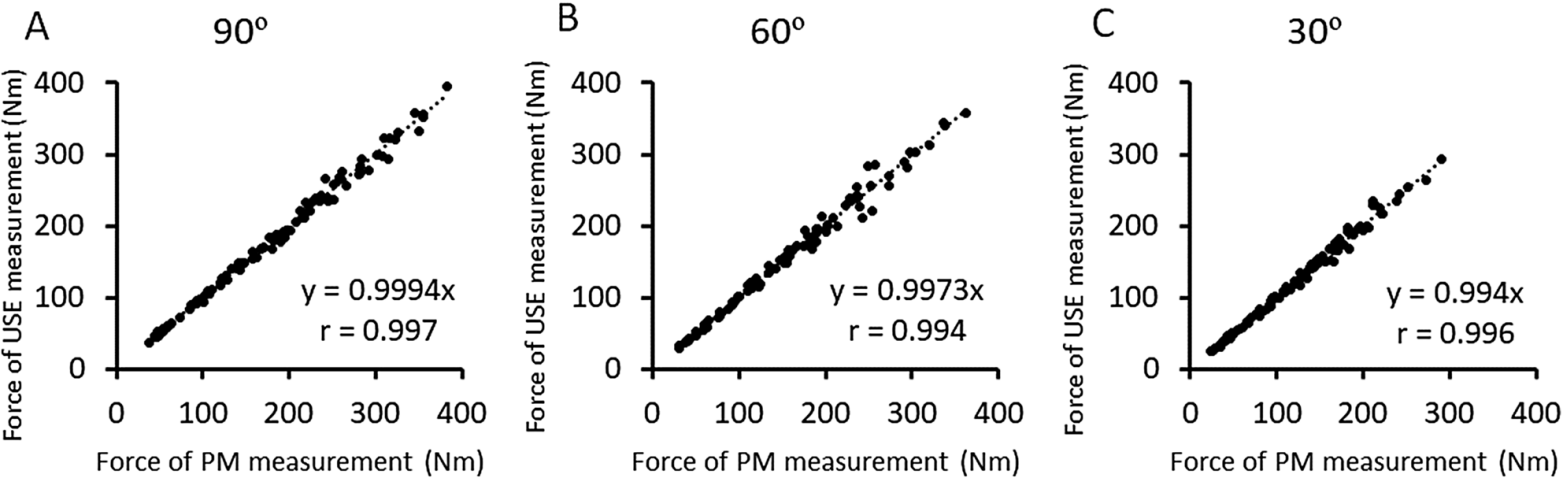
Relationships between push-in meter and ultrasound elastography measurements at the elbow joint angle of 90° (**A**), 60° (**B**), and 30° (**C**) for the muscle force of the elbow flexors (n = 16). High correlations in the elbow flexion muscle force between the measures are seen in all angles (90°: r = 0.997, 60°: r = 0.994, 30°: r = 0.996).

First, each participant was asked to flex the elbow joint maximally to pull the turnbuckle toward the right shoulder for 5 s. The maximum force of 3 trials was used as maximum voluntary contraction (MVC) force. Then, six levels of muscle contractions; 15%, 30%, 45%, 60%, 75% and 90% of MVC force were performed in this order with one-minute rest between contractions at the same intensity twice, and two-minute rest between different intensities. A target force was displayed on a computer screen, and each contraction lasted for 5 s. During the contraction, the push-in meter measurement and the elastography measurement were performed alternatively (one for each) at the measurement point. This procedure was done for three different elbow joint angle; 90°, 60° or 30° (full extend is 0°) in this order. It was made sure that the muscle force produced in the PM and USE measures was the same. Figure 2 shows the relationship between PM and USE measurements at elbow joint angle of 90°, 60° and 30°, respectively for the muscle forces. The forces were similar between the measures for all angles (90°: r = 0.997, 60°: r = 0.994, 30°: r = 0.996). Therefore, it was assumed that BB was activated similarly in the PM and USE measurements.

### Push-in meter (PM)

Figure 1A shows the push-in meter (PM) set-up for biceps brachii muscle hardness measure. The depth of deformation and the reaction force from the muscle were recorded using the PM system. This PM system was described in detail in a previously published paper^19^. Briefly, based on a two-layered spring model by Horikawa et al.^3^, the displacement-force curve was divided into subcutaneous and muscle component using a B-mode ultrasound image. The muscle hardness value was calculated by the equation: E = Id (1 − μ^2^) Km, where I is an influence coefficient, d is the diameter of the probe, μ is Poisson’s ratio and Km is the slope of the muscle component obtained from the force–displacement relationship. However, as the amount of distortion increases, the force–displacement relationship changes from linear to exponential with the slope being steeper. If muscle hardness is calculated with the same amount of distortion, it may overestimate the muscle hardness of an individual with a smaller muscle thickness. To avoid this, we calculated muscle hardness value (E) using the slope of the force curve ranged in 0–30% of a muscle thickness of each participant^19^. In the present study, the indentation of the tip of the probe was 20-mm from the surface. This amount of indentation was sufficient to measure a biceps brachii muscle hardness, because a maximum subcutaneous tissue thickness was 5.6 mm, and 30% muscle thickness of biceps brachii was 5.2–9.4 mm among the participants.

### Ultrasound strain elastography (USE)

**Figure 3:**
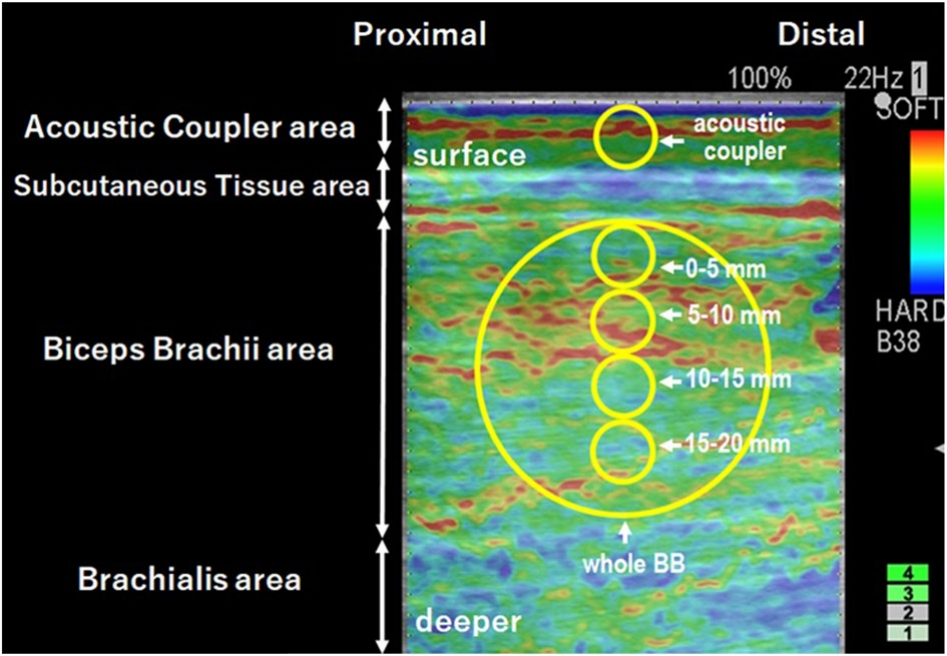
An ultrasound strain elastography image with an acoustic coupler taken from the biceps brachii muscle (BB). The region of interest (ROI) was set for acoustic coupler area (5-mm in diameter) and a circle including the whole BB (large circle) and a circle with a diameter of 5 mm from shallow to deep regions. First to 4th ROIs (0–5 mm, 5–10 mm, 10–15 mm, 15–20 mm) and whole BB were used for analysis, then ROIs deeper than 20 mm were excluded from analysis because of small number of data.

Transverse axial B-mode images of the biceps brachii muscle were obtained by a ultrasound system (Prosound F75; Hitachi Aloka Medical, Japan). Elastograph images were recorded while gently pressing a transducer with a reference material (acoustic coupler, YM = 22.6 kPa: EZU-TECPL1, Hitachi Aloka Medical Japan) over biceps brachii mid-belly. The investigator manually pressed the transducer against the muscle with rhythmical compression-relaxation cycles to provide consistent pressure speed^14^. While monitoring the strain level to be 1–4 as shown in the system screen (bottom right of Fig. 3), the transducer was pressed to the level of 2 or 3. In addition, care was taken to ensure that the transducer angle was always perpendicular to the muscle belly, where was indicated by a mark on the skin.

A circular region of interest (ROI) was set in the coupler and the muscle under the mark on the skin (Fig. 3). For analysis of SR distribution, a circular (ϕ = 5 mm) region of interest (ROI) was set in the coupler and the muscle (Fig. 3). Since previous studies have adopted ROI that occupy a large area of target muscle^11,16,20^, the present study also set a ROI for entire biceps brachii (whole BB, ϕ ≒ 20–30 mm). In addition, a small circular ROI (ϕ = 5 mm) was set at every 5-mm layer from surface to deep muscle (0–5 mm, 5–10 mm, 10–15 mm and 15–20 mm). As shown in Fig. 3, the hardness was not spatially uniform, but the region of interest (ROI) was always set at the image under the center of the transducer, and this was consistent over the measures.

Using a built-in software, strain ratio (SR) was calculated for each image as a ratio of strain of the muscle divided by the strain of the acoustic coupler (22.6 kPa). When SR is 1.0, the muscle hardness is considered to be identical to the reference (22.6 kPa). If SR is smaller than 1.0, muscle is harder than the coupler (> 22.6 kPa), thus it was expected that SR would get smaller in muscle contraction. For instance, if a muscle becomes 2 times and 10 times harder than the coupler, the SR shows 0.5 and 0.1, respectively. Thus, SR is a non-linear function and it does not fall below 0. In order to analyze the correlation between SR (non-linear) and the muscle hardness assessed by PM (linear), the SR value was logarithmically converted so that it can be treated as data showing a linear distribution in the present study. In this case, logarithmical transformed SR (LTSR) is 0 when SR is 1.0, LTSR is − 0.3 when SR is 0.5, and LTSR is − 1 when SR is 0.1, for example.

SR shows the muscle hardness from the relative relationship with the coupler (YM = 22.6 kPa). Muscle hardness has not been expressed as YM in previous studies using USE, but it is possible that YM of the muscle can be obtained from the SR. If muscle hardness obtained from USE and PM can be compared by YM, it is better to clarify the relationship between them better. Therefore, the present study converted SR to YM from the known Young’s modulus of the coupler by the formula; 22.6/SR. Thus, if SR was 0.5, its converted YM was 45.2 kPa, and if SR was 0.1, YM was 226 kPa, for example.

### Statistical analysis

SPSS version 24.0 was used to perform statistical analyses of the collected data. Data of E and SR calculated from each ROI were screened for normality and homogeneity using the Shapiro-Wilk and Levene’s test, respectively. One-way ANOVA was used to compare SR among different ROIs, and Dunnett’s T3 post hoc test was applied, because homogeneity of data was not assumed. Pearson’s correlation analyses were performed between E and LTSR or converted YM, E and MVC level, and LTSR and MVC level. A significance level was set at *p* < 0.05. All values are expressed as mean ± SD.

## Results

### Experiment 1: Resting condition

**Figure 4:**
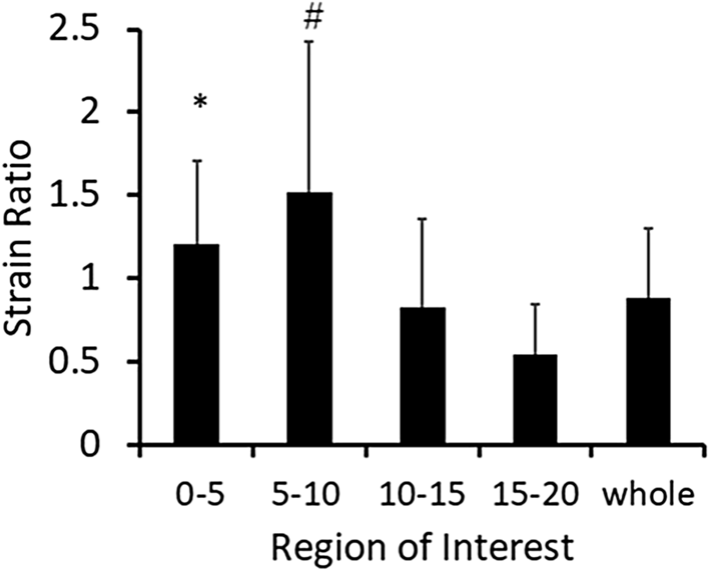
Comparison (mean ± SD values) of strain ratio (SR) for different regions of interest (ROI): 0–5, 5–10, 10–15, 15–20 mm and whole BB. SR in 5–10 mm was significantly larger (*p* < 0.05) than SR in 10–15 mm, 15–20 mm and whole BB (show by *). SR in 0–5 mm was significantly (*p* < 0.05) larger than SR in 15–20 mm (show by #).

Muscle hardness (E) assessed by PM was 26.1 ± 6.4 (range 15.0–40.4) kPa. Figure 4 shows the SR of five different ROIs: 0–5, 5–10, 10–15, 15–20 mm and whole BB. A significant difference was found between 5 and 10 mm and 10–15 mm, 15–20 mm as well as whole BB, and between 0–5 mm and 15–20 mm, indicating that the 5–10 mm region was softer than the 10–15 mm, 15–20 mm and whole BB, and the 0–5 mm region was also softer than the 15–20 mm region. Mean ± SD value of SR in the 5–10 mm was 1.52 ± 0.9 (converted YM: 20.2 ± 11.5 kPa), and those in the 15–20 mm and whole BB were 0.55 ± 0.3 (54.9 ± 27.3 kPa) and 0.88 ± 0.4 (30.8 ± 12.8 kPa), respectively. A dependent t-test showed that the converted YM of the whole BB (30.8 ± 12.8 kPa) was significantly (*p* = 0.048) greater than the muscle hardness assessed by E (26.1 ± 6.4 kPa), but the two values were significantly correlated (r = 0.615, *p* = 0.003).

**Figure 5:**
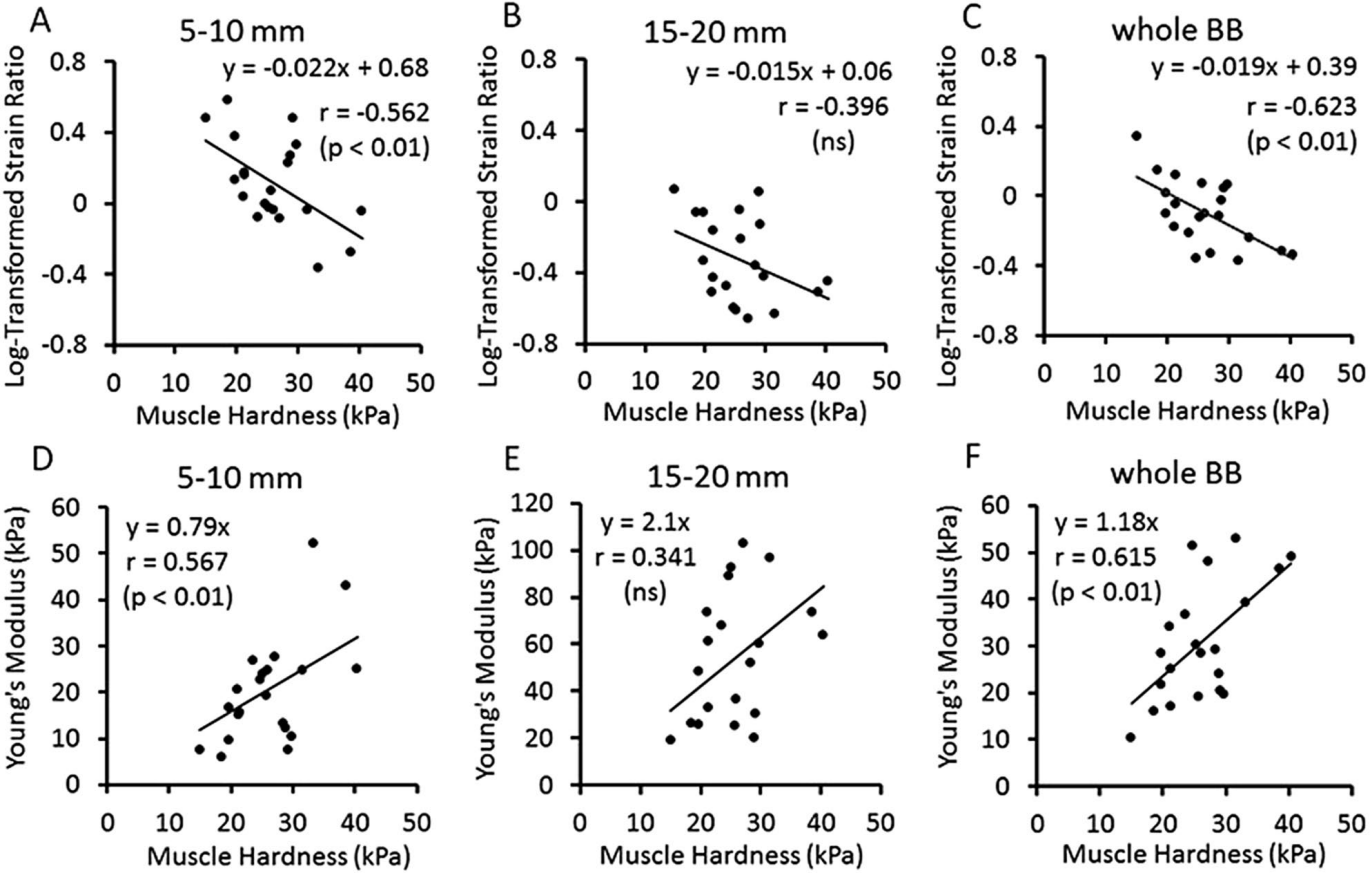
Relationships between muscle hardness (E: Young’s modulus assessed by push-in meter) and logarithmical transformed strain ratio (LTSR) (**A**–**C**) or E and Young’s modulus converted from SR (YM) by 22.6 kPa/SR (**D**–**F**) for the ROI of 5–10 mm, 15–20 mm and whole biceps brachii (BB) in resting condition. Significant (*p* < 0.01) correlations between E and LTSR were evident for whole BB (r = − 0.623, **C**) and 5–10 mm ROI (r = − 0.562, **A**). E and YM from SR were significantly (*p* < 0.01) correlated for the 5–10 mm ROI (r = − 0.567, **D**) and for the whole BB (r = 0.615, **F**) with the regression coefficient was close to 1, y = 1.18 x.

Figure 5 shows the relationships between E and LTSR (A-C) and between E and YM converted from SR (D-F) for the ROI of 5–10 mm, 15–20 mm and whole BB. Significant correlations (Pearson’s r) were evident between E and LTSR or E and YM for the 5–10 mm ROI and whole BB. The regression equation for the relationship between E (x) and YM (y) for the whole BB was y = 1.18 x.

### Experiment 2: Muscle contraction condition

**Figure 6:**
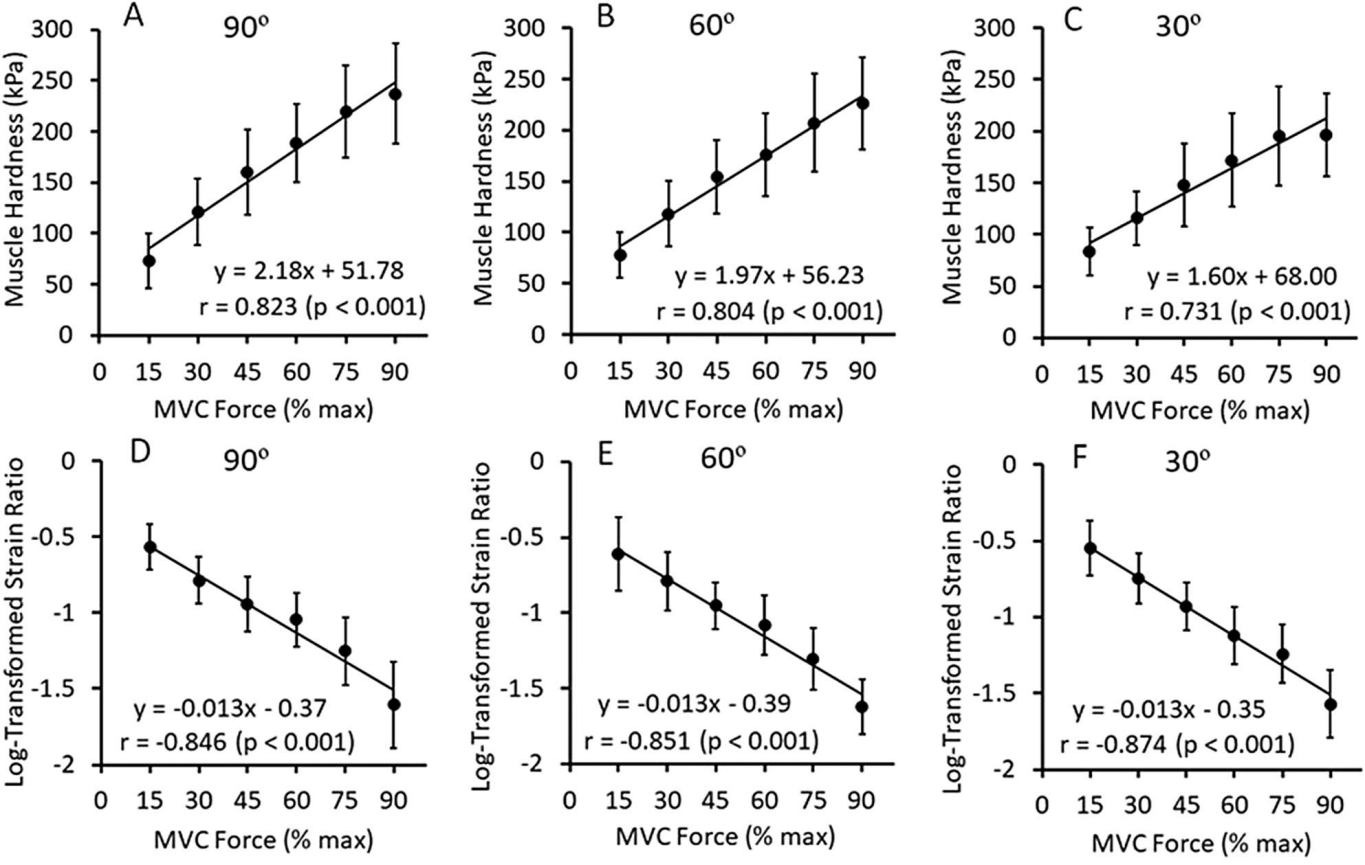
Relationships between force level relative to maximum voluntary contraction (MVC) force and muscle hardness assessed by push-in meter (E: Young’s modulus) (**A**–**C**) or logarithmically transformed SR (LTSR) (**D**–**F**) for whole biceps brachii at the elbow joint angle of 90°, 60°, and 30°. Values are mean ± SD (n = 16), and a linear regression line was fitted for all data. Significant (*p* < 0.001) correlations were found for relative force level to MVC and E (90°: r = 0.823, 60°: r = 0.804, 30°: r = 0.731) as well as LTSR (90°: r = − 0.846, 60°: r = − 0.851, 30°: r = − 0.874).

Figure 6 shows the relationship between the intensity of MVC force and E (A–C) or LTSR (D–F) at elbow joint angle of 90°, 60° and 30°, respectively. For LTSR, the whole BB was selected because the correlation with E was the highest for the resting muscle as shown in Fig. 5. E values increased linearly with increasing in the muscle contraction intensity at each elbow joint angle. When comparing the r values among the three angles, the r value was the highest at 90° followed by 60° then 30°. At 90°, E at 90% MVC was 237.1 ± 49.3 kPa (range 154.9–309.5 kPa), which was approximately ninefold greater than that at resting condition (26.1 ± 6.4 kPa). On the other hand, LTSR decreased linearly with increasing the MVC level, and the relationship was similar among the three elbow joint angles. The range of LTSR was − 0.5 to − 1.5 (SR was 0.32 to 0.032), indicating 2.8-fold to 28-fold increase in muscle hardness from the resting condition (SR = 0.88).

**Figure 7:**
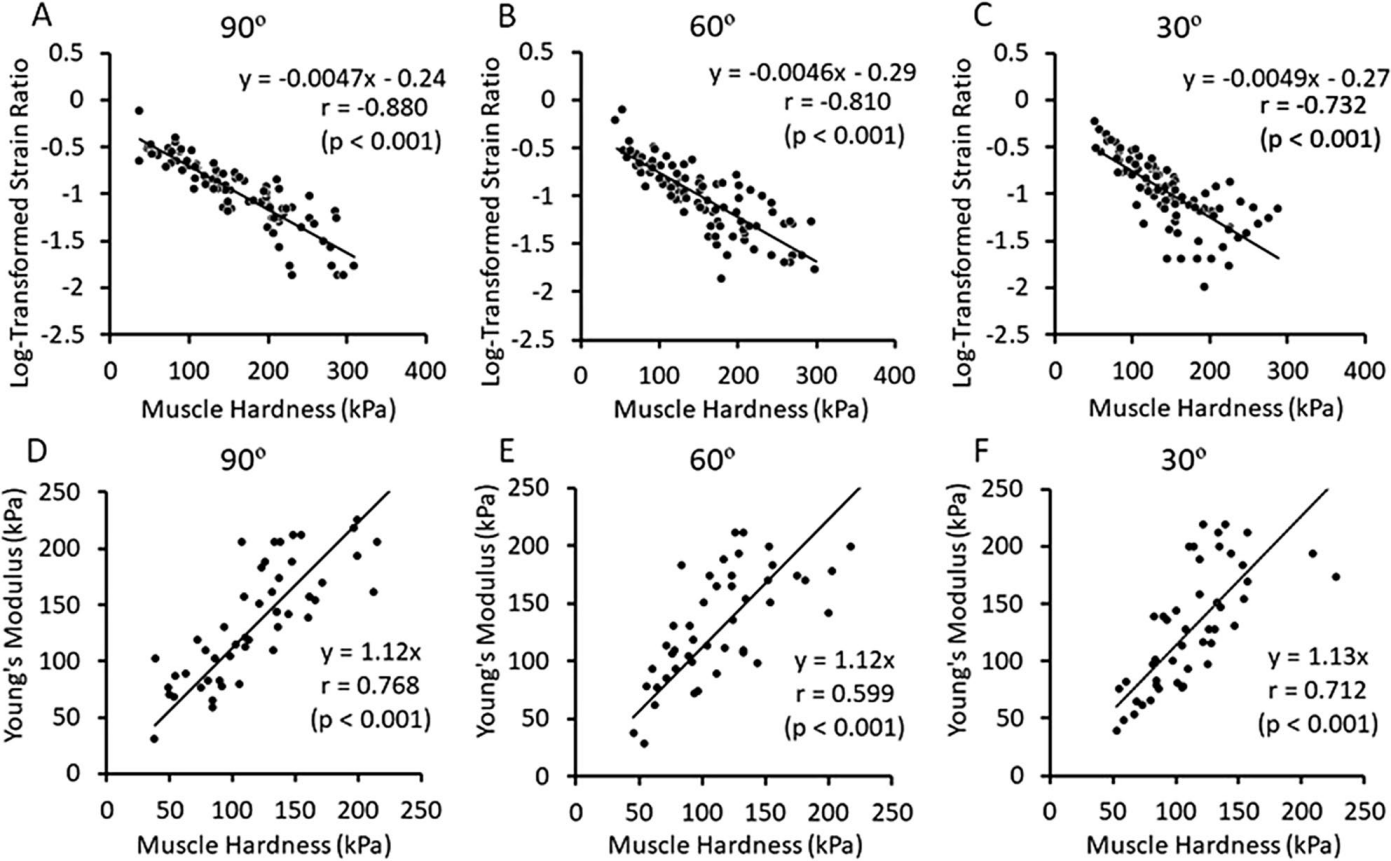
Relationships between muscle hardness assessed by a push-in meter (E: Young’s modulus) and logarithmical transformed strain ratio (LTSR) (**A**–**C**) or Young’s modulus (YM) converted from SR (22.6 kPa/SR) (**D**–**F**) in whole biceps brachii for different muscle contraction intensities (15–90% of maximal voluntary contraction force) at the elbow joint angle of 90°, 60°, and 30°. Significant correlations were observed between E and LTSR for all joint angles (90°: r = − 0.880, 60°: r = − 0.810, 30°: r = − 0.732) (**A**–**C**). Significant correlations were also evident between E and YM for all joint angles (90°: r = 0.768, 60°: r = 0.599, 30°: r = 0.712) with the regression coefficient was close to 1 (90°: y = 1.12 x, 60°: y = 1.12 x, 30°: y = 1.13 x) (D-F).

Figure 7 shows the relationships between E and LTSR (A–C) or E and YM converted from SR (D–F) for the whole BB at the elbow joint angle of 90°, 60° and 30°, respectively. E was negatively correlated with LTSR for all angles, without a significant difference among the three angles. However, when E was greater than 200 kPa, the variation in LTSR became larger. Since E showed approximately ninefold increase from the resting to contracting condition at 90% of MVC, and the high correlation between E and SR shown in the resting muscle (Fig. 5), SR change was also considered to be approximately ninefold from the rest to 90% MVC. For this range; SR > 0.098 (1/9 of 0.88), E (x) and YM converted from SR (y) were correlated significantly for all elbow joint angles, and the regression coefficient was close to 1 (90°: y = 1.12 x, 60°: y = 1.12 x, 30°: y = 1.13 x).

## Discussion

The present study compared biceps brachii muscle hardness value E (Young’s modulus: YM) measured by PM and SR measured by USE for the resting (Experiment 1) and contracting (Experiment 2) conditions to test the hypothesis that E and SR would be significantly correlated. Considering that SR is a variable that changes in a fractional function, logarithmical transformed SR (LTSR) was used for the correlation analysis. SR was different among the ROIs at different depth, and the 5–10 mm region was softer than other ROIs (Fig. 4), but E value showed the highest correlation with the LTSR in the whole BB (Fig. 5). In contracting muscle, E increased and LTSR decreased linearly with increasing in MVC force for the three elbow joint angles (Fig. 6), and significant correlations between E and LTSR of the whole BB (Fig. 7A–C) as well as between E and YM converted from SR (Fig. 7D–F) were evident for the three elbow joint angles. These results supported the hypothesis and showed that muscle hardness assessment by means of PM and USE was comparable.

In the resting muscle, SR of the first and second ROIs (0–10, 5–10 mm) was larger (the ROIs were softer) than the deeper ROIs or whole BB (Fig. 4). This suggests that strain distribution was not homogeneous in a muscle, and the muscle at a deeper region is harder than that at a shallower region. It should be noted that the isotropicity of muscular tissue cannot be assumed^21,22^, and a size of the ROI in USE study is not standardized^23^. It was reported that SR was not influenced by depth of muscle^24^, and shear wave speeds at 1.5 cm depth and 2.0 cm depth were not different^25^. However, when a deeper tissue is deformed, the influence of the pressure from the upper layer is considered to be added. In the present study, the elbow joint was extended thus the biceps brachii muscle was stretched and dense, so it is possible that the deeper layers were likely to be less distorted. This is the first study to report SR of different ROIs based on the distance from the surface. Further research is needed to investigate strain distribution in a muscle.

It should be noted that the correlations between E and LTSR, as well as E and YM at resting condition were not high, although significant (*p* < 0.01) correlations were evident for the whole BB (r = − 0.623, r = 0.615) and 5–10 mm ROI (r = − 0.562, r = 0.567) (Fig. 5). This may be due to the difference in the magnitude of compression between PM and USE, such that PM pushes more than 30% of the muscle thickness, but less than 5 mm in USE. The coefficient of variation (CV) of strain measurement by USE was high (5–10 mm: 59.2%, 15–20 mm: 54.5%, whole BB: 45.4%), but that for the E measured by PM was smaller (24.5%). LTSR and YM also had a large variance, thus the correlation with E was weakened. It has been shown that the average strain of a muscle is better represented by a larger ROI^24^, thus a ROI should cover a large region of a target tissue^26^. Previous studies in which USE was used to assess muscle hardness used a ROI to cover a large area of a muscle^11,16,20^. It appears that the muscle hardness by SR from the whole BB than the smaller ROIs was more comparable to the muscle hardness assessed by the PM.

The biceps brachii muscle hardness values obtained in the present study by PM (15.0–40.4 kPa) were in line with those in which PM was used to assess biceps brachii muscle hardness reported by Murayama et al.^19^: 23.1 ± 6.5 kPa, and Komiya et al.^6^: 30.4 ± 8.2 kPa. To the best of our knowledge, no previous study has reported YM of biceps brachii assessed by USE, but some studies used ultrasound shear wave elastography (USWE) that can evaluate YM based on the shear wave velocity (V): YM = 3pV^2^ (*p* = density: 1 g/cm^2^)^27^. YM values of biceps brachii by USWE varied among the studies, but Akagi et al.^28^ reported 19.4 ± 6.8 kPa, and Nordez and Hug^29^ reported 33.9 ± 11.4 kPa, which were in the range obtained in the present study.

As shown in Fig. 5F, the relationship between E and converted YM from SR of the whole BB was y = 1.18 x, suggesting that the muscle hardness values derived by YM were 18% greater in average than those by E. The average muscle hardness (E) by PM measure was 26.1 ± 6.4 kPa, and that by USE in terms of YM was 30.8 ± 12.8 kPa. When looking at individual data for the paired comparison between the two values from PM and USE, 14 out of 21 participants showed a larger value for the USE than the PM measures, suggesting that muscle hardness by USE tends to be greater than that by PM. It should be noted that the PM determined E from the slope of the force–displacement relationship for the depth of 30% of muscle thickness in the present study, which was considered to induce a deformation of the muscle deeper than 30%. Since the Poisson's ratio of the muscle was assumed to be 0.5^3^ the lateral deformation is assumed to be 15% of the whole biceps brachii, thus the muscle deformation by PM extended over a range of 30% in depth and 10 mm in width. This was not exactly the same as the ROI set for USE to assess the muscle hardness. This may explain the difference in the muscle hardness values obtained by PM and USE. This also explains why E had a stronger relationship with the strain (SR) calculated from the whole BB than the four small ROIs set from the surface.

Regarding the contracting condition, E and LTSR changed linearly to the increase in the relative MVC, but in an opposite direction such that E increased while LTSR decreased (Fig. 6). Using isolated frog muscles, it has been demonstrated that the hardness measured by perpendicular indentation to the direction of muscle fiber is strongly correlated with the muscle tension generated by electrical stimulation^30^. In human muscles, a high positive linear correlation (R^2^ = 0.96) between muscle hardness measured by soft tissue stiffness meter and isometric force of the forearm extensors (0 – 100% MVC) was reported^31^. Muscle hardness assessed by PM increased 3.2-fold from 15% MVC (73.3 ± 26.6 kPa) to 90% MVC (237.1 ± 49.4 kPa). The previous studies showed that biceps brachii muscle hardness increased approximately threefold from 20% MVC to 80%MVC^7^ and approximately twofold from 15% MVC to 60% MVC^6^. Thus, it appears that changes in E value with changes in MVC force in the present study are in line with those reported in the previous studies.

Regarding the relationship between MVC intensity and LTSR, they were negatively but linearly correlated (Fig. 6D–F). When muscle force increased from 15% MVC to 90% MVC, LTSR decreased from − 0.57 ± 0.15 (SR: 0.29 ± 0.13) to − 1.61 ± 0.28 (SR: 0.03 ± 0.02), indicating approximately tenfold increase in muscle hardness, because SR became approximately one-tenths from 0.29 to 0.03. Furthermore, E was correlated with LTSR significantly at all elbow joint angles (Fig. 7A–C). These results suggest that changes in muscle harness were detected similarly by PM and USE. However, it should be note that some of the LTSR values were − 1.5 or less at above 75% MVC (Fig. 7A–C). This means that muscle hardness increased by more than 30-fold from resting to high-intensity contraction in the muscle hardness assessment by USE. It appears that SR overestimates muscle hardness when a muscle is hard (e.g., above 75% MVC), as the saturation of SR during contraction was pointed out previously^11^. It may be that a harder reference coupler (e.g., 50 kPa) is necessary to assess a harder muscle.

When the relationship between E and YM converted from SR was limited to ninefold change from the resting to contracting condition, a significant correlation was found between the two, and the regression equation was similar to the resting condition (y = 1.18 x), for all elbow joint angles (Fig. 7D–F). These results suggest that the relationship between E and the YM in the contacting muscle was similar to that of resting muscle, and the muscle hardness by YM (USE) was 12–13% greater in average than that by E (PM) for the contracting muscle, which was not much different from the resting muscle showing that the muscle hardness assessed by USE was 18% in average greater than that by PM. PM and USE are likely to assess the same mechanical properties of the muscle in a limited range that is considered to be in physiological ranges. The present study was the first to report the relationship between PM and USE for muscle hardness assessment, and showed that a correlation coefficient for the biceps brachii muscle hardness in terms of Young’s modulus was significant between PM and USE for both resting and contracting conditions.

Comparing PM and USE in the present study, the reliability of PM is considered to be better, because the CV of SR was large, and the LTSR at high intensity contraction showed unreasonable value (28-fold increase from the resting value). It seems possible that PM provides more accurate muscle hardness values than USE. The PM system used in the present study overcomes the inadequacy of the hand-held type PM by providing a sufficient indentation and using a two-layer spring model^19^. Bilston and Tan^21^ stated that myotonometry (one of the PM) could assess muscle mechanical properties such as tissue compliance in patients with spasticity and neuromuscular disorders just like an elastographic method with low cost. The results of this study are the basis for further recommending the use of PM.

USWE seems to be used more than USE to assess muscle hardness or stiffness in recent years. It is interesting to compare PM and USWE for muscle hardness in a similar way to that of the present study. In the present study, only biceps brachii was investigated, but other muscles should be investigated in future studies. It is also important to compare muscle hardness assessed by USE or USWE and PM for clinical conditions such as stiff shoulder or muscle diseases. The PM measurement system in the present study cannot be customized to combine with the measurement of USE. It is difficult to obtain a hardness or strain distribution like an elastography from the PM. Since an ultrasound machine with elastography is expensive and is not necessarily convenient in practical sports and clinical settings, development of a simple, inexpensive and accurate PM such as a hand-held device is required. Several studies have developed muscle hardness measure devices that can also monitor ultrasonic images^32,33^. We have also developed a system with a force transducer built into an ultrasound probe^34^. It may be possible to combine the PM and USE measures in the future.

In conclusion, the results of the present study showed that muscle hardness values obtained by PM and USE were highly correlated for resting and contracting biceps brachii, and suggest that PM and USE indicate muscle hardness and its changes similarly. Thus, it seems that PM basically evaluates muscle hardness similarly to that by USE. Muscle hardness assessment using PM should be developed further for functional evaluation of various muscles in various situations, since it appears to be valid method against the methods by ultrasonography to examine muscle hardness and its changes, since PM is cheaper and more convenient in field studies and practical settings.
